# Intolerable side effects during propranolol therapy for infantile hemangioma: frequency, risk factors and management

**DOI:** 10.1038/s41598-018-22787-8

**Published:** 2018-03-09

**Authors:** Yi Ji, Siyuan Chen, Qi Wang, Bo Xiang, Zhicheng Xu, Lin Zhong, Kaiying Yang, Guoyan Lu, Liqin Qiu

**Affiliations:** 1 Division of Oncology, Department of Pediatric Surgery, West China Hospital of Sichuan University, Chengdu, 610041 China; 2 Pediatric Intensive Care Unit, Department of Critical Care Medicine, West China Hospital of Sichuan University, Chengdu, 610041 China; 3 Pediatric Intensive Care Unit, West China Second University Hospital, Sichuan University, Chengdu, 610041 China; 4 Department of Pediatric Surgery, Chengdu Shangjin Nanhu Hospital, Chengdu, 611730 China

## Abstract

Currently, propranolol is the most preferred systemic therapy for problematic infantile hemangiomas (IHs). However, the side effects such as bronchial hyperreactivity may be intolerable. The aim of this study was to evaluate the frequency, risk factors and management of intolerable side effects (ISEs) during propranolol therapy. In total, 1260 children were studied. The incidence of ISEs was 2.1% (26 patients). Severe sleep disturbance was the most common reason for propranolol cessation, accounting for 65.4% of cases. In total, 23 and 3 patients received atenolol and prednisolone as second-line therapy, respectively. Treatment response was observed in 92.3% (24/26) of cases (showing excellent or good response to therapy). No toxicity-related permanent treatment discontinuation occurred during atenolol or prednisolone therapy. In the univariate analysis, younger age, premature birth, and lower body weight were associated with ISEs (*P* < 0.05). In the multivariate analysis, only age (95% confidence interval [CI]: 1.201–2.793, *P* = 0.009) and body weight (95% CI: 1.036–1.972, *P* = 0.014) were associated with ISEs. Our study suggests that ISEs are rare in patients with IHs who are treated with propranolol. Predictive factors for ISEs include younger age and lower body weight. Atenolol and prednisolone are effective and safe alternatives to propranolol in the treatment of refractory IHs.

## Introduction

Infantile hemangiomas (IHs) are the most common benign vascular tumor in children with an estimated prevalence of 5–10%. Although most IHs resolve spontaneously without threat or complication, approximately 12–24% of IHs have complications and require treatment. In severe cases, early treatment is warranted to arrest the growth of the IH, reduce potential complications, avoid psychosocial concerns, and improve quality of life^1,2^.

Propranolol, a lipophilic nonselective β-blocker, is now introduced as first-line treatment for IHs requiring systemic therapy. Although propranolol has clearly been efficacious, side effects have occasionally been reported during treatment^3^. The high liposolubility of propranolol can facilitate its passage from the blood to the brain. As a result, patients may have a higher risk of side effects related to the central nervous system (CNS) (e.g., sleep disturbance and agitation). In addition, serious side effects such as bronchospasm/bronchial hyperreactivity and hypoglycemia are direct effects of β_2_-adrenergic receptor (β_2_-AR) blockade caused by propranolol^4^. Some patients must discontinue treatment due to intolerable side effects (ISEs), resulting in a higher incidence of rebound growth of the tumors^5^.

The goal of the present study was to evaluate the frequency, risk factors and management of ISEs during propranolol treatment.

## Methods

We conducted a retrospective review from August 2013 to January 2016. Data were collected on patients who were treated for problematic IHs with propranolol. Approval was obtained from the West China Hospital of Sichuan University Institutional Review Board, the study site of the principal investigator, and by the local institutional review boards at each participating site. All procedures followed the research protocols approved by Sichuan University and West China Hospital of Sichuan University and was conducted according to the Declaration of Helsinki. Written informed consent was obtained from all patients’ parents. Patients were excluded if they had insufficient data. Patients who withdrew from propranolol treatment due to a lack of efficacy and/or parents’ consent were also excluded.

Before treatment, patients’ parents provided a thorough medical history (e.g., existence of comorbidities) and family history (e.g., cardiovascular disease). A Physical examination and baseline electrocardiogram (ECG) were performed on all infants. Propranolol was initiated at a dosage of 1.0 mg/kg per day, which was divided into 3 daily administrations for 1 week, and then, starting at week 2, the dosage increased to 2.0 mg/kg per day, which was divided into 3 daily administrations. During treatment, the doses were adjusted for weight gain.

Patients’ demographics, clinical presentation, physical findings and laboratory results were assessed at baseline, at the initiation of each treatment regimen, at each scheduled visit, and at the last available visit. Photographs of hemangiomas were taken at weeks 0 and 24 and were assessed by using the Hemangioma Activity Score. The outcomes were classified as deterioration (further growth of the IH), stable (no change), good (partial involution) or excellent (compete or nearly complete involution) at week 24 versus baseline according to the evaluation.

Data on ISEs were collected and graded according to the Common Terminology Criteria for Adverse Events, version 4.0 (CTCAE v4.0). The causality of the side effects during propranolol treatment was assessed by the investigators and determined by the relationships among time to drug intake, effect of dechallenge or rechallenge of drugs, or absence of other diseases^6^. The relationships were classified as definitively not related, probably not related, possibly related, probably related, or definitively related. Only patients with side effects that were at least possibly treatment-related were taken into consideration^7^. Mild symptoms might have subsided without any interventions, or they often resolved when the treatment administration was altered (e.g., earlier evening dose or a decrease in daily dose). The ISEs were adverse events needing discontinuation of propranolol administration.

Statistical analyses of in the study were conducted using SPSS 22.0 for Windows (SPSS Inc, Chicago, USA). A Pearson’s chi-squared test and Fisher’s exact test were used to analyze of categorical variables. Multivariate logistic regression analyses were performed to detect the independent risk factors for sleep disturbances with odds ratios (ORs) and 95% confidence intervals (CIs). *P* values less than 0.05 were considered significant.

## Results

### Patient demographics and IH characteristics

Data from 1260 patients from 3 individual centers were collected. The baseline characteristics of patients are summarized in Table 1. There were 318 males and 942 females, with a male to female ratio of 1:2.96. Of the 1260 patients, 226 (17.9%) were born prematurely. The median age at the start of propranolol therapy was 96 days (interquartile range [IQR], 68–155 days). The patients’ median weight at the time of propranolol initiation was 6.4 kg (IQR, 5.9–6.8 kg). In total, 53.0% of IHs were located on the head and neck. The most frequently observed morphologic and description subtypes were localized (70.0%) and mixed (74.8%), respectively. Propranolol was administered for a median duration of 337 days (IQR, 249–423 days) (Table 1).

**Table 1:** Characteristics of patients and His.

Characteristics	Total patients	Patients’ reported intolerable side effects
**Patients**		
Gender^†^		
Male	318 (25.2)	7 (26.9)
Female	942 (74.8)	19 (73.1)
Gestational age^†^		
Term born (≥37 w)	1034 (82.1)	18 (69.2)
Born prematurely (<37 w)	226 (17.9)	8 (30.8)
Age when starting propranolol treatment (d)^‡^	96 (68–155)	60 (49–77)
Body weight when starting propranolol treatment (kg)^‡^	6.4 (5.9–6.8)	5.7 (5.2–6.1)
Duration of propranolol treatment (d)^‡^	337 (249–423)	50 (18–85)
**IHs**		
Location^†#^		
Head, face and neck	668 (53.0)	14 (53.8)
Extremity	211 (16.7)	4 (15.4)
Trunk	326 (25.9)	7 (26.9)
Perineal	45 (3.6)	1 (3.8)
Internal	10 (0.8)	0 (0)
Morphologic subtype^†^		
Localized	882 (70.0)	18 (69.2)
Segmental	125 (9.9)	3 (11.5)
Indeterminate	201 (16.0)	4 (15.4)
Multifocal	52 (4.1)	1 (3.8)
Description^†#^		
Superficial	190 (15.1)	4 (15.4)
Mixed	943 (74.8)	20 (76.9)
Deep	127 (10.1)	2 (7.7)

^*^IHs, infantile hemangioma; d, day; w, weeks.

^†^Values are presented as a number (percentage).

^‡^Values are presented as a median (interquartile range).

^#^In multiple IHs, only the clinically most important IH (typically the largest or ulcerated IH) was documented.

### Intolerable side effects

Of the 1260 patients, 26 patients (2.1%) experienced ISEs that were identified by the investigators as being at least possibly treatment-related (Table 2). Seventy-three percent of these side effects appeared within the first 30 days of propranolol treatment. In all patients, propranolol was eventually discontinued. The median duration of propranolol therapy in these patients was 50 days (IQR, 18–85 days).

**Table 2:** Characteristics of 26 IH patients with intolerable side effects during propranolol treatment.

Cases	Sex	Location	Age at initiation of propranolol (d)	Morphologic subtype	Description	Duration of propranolol treatment (d)	Reason for discontinuation	Two treatment Interval (d)	Following treatment	Response
1	Female	Head	46	Localized	Mixed	30	Sleep Disturbance	7	Atenolol	Excellent
2	Female	Face	42	Segmental	Deep	120	Sleep Disturbance	0	Atenolol	Excellent
3	Female	Face	102	Localized	Mixed	12	Sleep Disturbance	2	Atenolol	Excellent
4	Male	Trunk	80	Localized	Mixed	60	Hypoglycemia	14	Prednisolone	Good
5	Female	Head	61	Localized	Superficial	21	Agitation	0	Atenolol	Excellent
6	Female	Perineal	66	Localized	Mixed	150	Sleep Disturbance	90	Atenolol	Good
7	Male	Face	40	Indeterminate	Mixed	4	Bronchial hyperreactivity	31	Prednisolone	Excellent
8	Male	Neck	72	Localized	Mixed	60	Sleep Disturbance	0	Atenolol	Excellent
9	Female	Extremity	60	Multifocal	Mixed	42	Bronchospasm	28	Atenolol	Good
10	Female	Trunk	49	Localized	Mixed	77	Sleep Disturbance	35	Atenolol	Excellent
11	Female	Face	35	Indeterminate	Superficial	55	Sleep Disturbance	1	Atenolol	Excellent
12	Male	Extremity	66	Indeterminate	Mixed	240	Sleep Disturbance	0	Atenolol	Stable
13	Female	Face	52	Localized	Deep	18	Agitation	7	Atenolol	Excellent
14	Female	Head	29	Localized	Mixed	6	Sleep Disturbance	21	Atenolol	Good
15	Female	Trunk	55	Segmental	Mixed	98	Sleep Disturbance	0	Atenolol	Excellent
16	Female	Face	66	Localized	Mixed	5	Hypoglycemia	7	Atenolol	Good
17	Male	Trunk	91	Localized	Mixed	120	Sleep Disturbance	7	Atenolol	Excellent
18	Female	Neck	80	Localized	Mixed	60	Sleep Disturbance	3	Atenolol	Excellent
19	Female	Extremity	49	Localized	Mixed	6	Bronchial hyperreactivity	14	Prednisolone	Excellent
20	Male	Head	55	Localized	Superficial	25	Sleep Disturbance	0	Atenolol	Good
21	Female	Trunk	92	Localized	Mixed	18	Sleep Disturbance	3	Atenolol	Excellent
22	Female	Face	80	Indeterminate	Mixed	42	Agitation	7	Atenolol	Excellent
23	Female	Trunk	48	Localized	Mixed	140	Sleep Disturbance	2	Atenolol	Good
24	Female	Extremity	60	Localized	Mixed	22	Bronchospasm	21	Atenolol	Stable
25	Male	Trunk	76	Segmental	Mixed	81	Sleep Disturbance	0	Atenolol	Excellent
26	Female	Face	52	Localized	Superficial	75	Sleep Disturbance	1	Atenolol	Excellent

^*^d, days.

Severe sleep disturbances were the most common reason for the cessation of propranolol, representing 65.4% (17/26) of all ISEs (Table 3). Three patients (11.5%) experienced severe agitation. All these symptoms persisted for more than 1 week with the administration of propranolol and affected the patients’ and/or parents’ quality of life.

**Table 3:** Intolerable side effects graded as possibly related to propranolol treatment.

Events	All grades	Grade 2	Grade 3	Grade 4
Sleep disturbance	17	3	14	—
Agitation	3	1	2	0
Bronchial hyperreactivity	2	0	0	2
Bronchospasm	2	0	1	1
Hypoglycemia	2	0	2	0

^*^Adverse events were assessed using the Common Terminology Criteria for Adverse Events, version 4.0.

Four patients (15.4%) reported severe respiratory disorders. Of these patients, 2 reported severe bronchial hyperreactivity, which occurred within 10 days after propranolol introduction. Bronchial hyperreactivity was considered life-threatening and required emergency airway management. Serious symptoms led to permanent discontinuation of propranolol treatment. Another 2 patients had bronchospasm associated with viral infection. Both had rapid resolution of wheezing after the discontinuation of propranolol.

Two patients experienced symptomatic hypoglycemia. One case had severe ulcerated IHs that interfered with normal oral intake. Five days after the initiation of propranolol, she experienced lethargy and was unresponsive in the morning. Her blood glucose was measured by paramedics to be 2.1 mmol/L. Another patient had concurrent viral gastroenteritis that was associated with vomiting and diarrhea. Both recovered after glucose perfusion.

### Management

The interval between the cessation of propranolol and the initiation of the following intervention ranged from 0 to 90 days. Rebound growth of the hemangioma after propranolol discontinuation was noted in 9 patients (31.0%). Of the 26 patients, 23 patients received oral atenolol treatment. Atenolol was initiated at a dosage of 0.5 mg/kg per day in a single administration for 1 week and then increased to 1 mg/kg per day in a single administration starting at week 2. In all patients, atenolol was administered in the morning and within 30 minutes after the patients were fed. After 24 weeks of treatment, an ‘excellent’ response was observed in 15 patients (65.2%), ‘good’ in 6 (26.1%), and ‘stable’ in 2 (8.7%).

The remaining 3 patients received a single daily dose of orally administered prednisolone (2 mg/kg per day). Of these 3 patients, 2 were reported to have an excellent response and 1 was reported to have a good response after 24 weeks of treatment.

**Figure 1:**
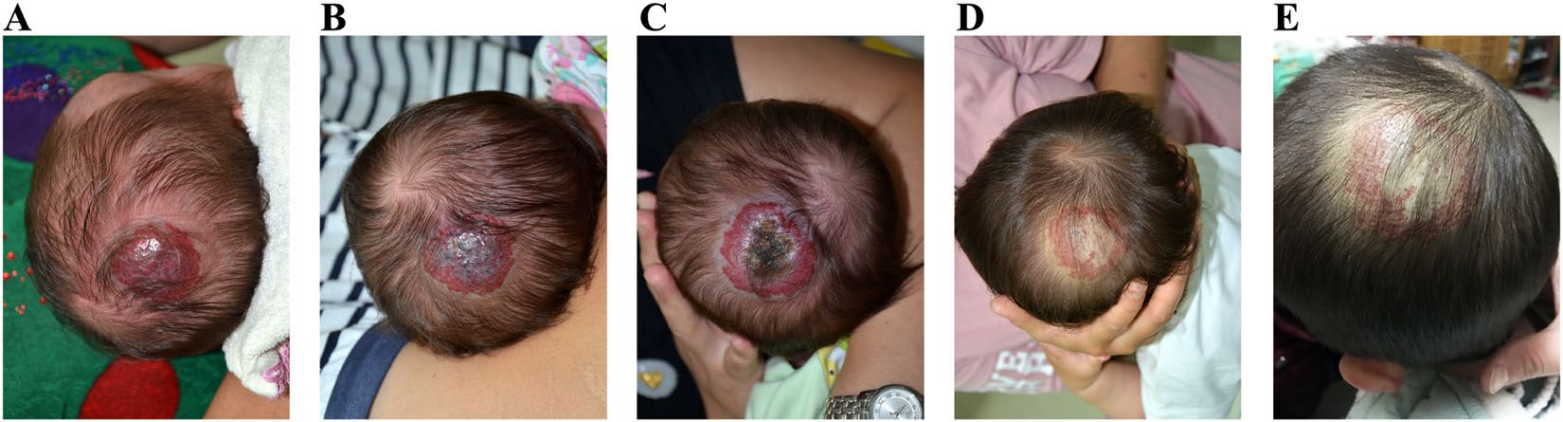
A 29-day-old girl with mixed infantile hemangioma (IH) on the vertex of the scalp. Clinical photograph of IH: (**A**) 1 day before treatment with propranolol. (**B**) 6 days after the start of propranolol. On the same day, the propranolol therapy was permanently discontinued due to severe sleep disturbance and agitation; (**C**) 3 weeks after the discontinuation of the propranolol therapy; the photograph shows that the rapid expansion of the lesion resulted in an ulceration with an overlying crust. Then, atenolol treatment was administered; (**D**) 24 weeks after atenolol treatment; (**E**) 48 weeks after atenolol treatment.

Atenolol and prednisolone were generally well tolerated in the patients who were previously intolerant to oral propranolol. No toxicity-related permanent treatment discontinuation occurred during atenolol or prednisolone therapy (Fig. 1).

### Risk factors

As shown in Table 3, a univariate analysis was performed to analyze the risk factors for ISEs. We found that age, gestational age, and lower body weight were significant factors associated with ISEs (*P* < 0.05) (Table 4). Based on the statistically significant difference shown in the univariate analysis, the results of a multivariate regression analysis indicated that age (95% CI: 1.201–2.793, *P* = 0.009) and body weight (95% CI: 1.036–1.972, *P* = 0.014) were independent risk factors for ISEs. In contrast, gestational age failed to reach independent significance in the multivariate analysis (*P* = 0.170) (Table 5).

**Table 4:** Risk factors for the intolerable side effects derived from univariate analysis.

Variable	With intolerable side effects (n = 26)	Without intolerable side effects (n = 1234)	*P*-values^†^	95% Confidence Interval
Age (d)*			0.001	1.838–20.637
<90	23 (88.5)	595 (48.2)		
90–180	3 (11.5)	478 (38.7)		
å 180	0 (0)	161 (13.0)		
Gender			0.843	0.455–2.626
Male	7 (26.9)	311 (25.2)		
Female	19 (73.1)	923 (74.8)		
Gestational age			0.035	1.092–5.640
Born prematurely	9 (34.6)	217 (17.6)		
Term born	17 (65.4)	1017 (82.4)		
Body weight (kg)*			0.024	1.103–9.889
<5	4 (15.4)	60 (4.8)		
5–10	22 (84.6)	1090 (88.5)		
å 10	0 (0)	82 (6.6)		

*Age when starting propranolol treatment; body weight when starting propranolol treatment.

^†^The differences are statistically significant if *P* < 0.05.

**Table 5:** Multivariate regression analysis to identify risk factors associated with intolerable side effects.

Variables	Odds ratio	*P*-values*	95% Confidence Interval
Age	1.974	0.009	1.201–2.793
Gestational age	0.079	0.170	1.056–1.671
Body weight	1.525	0.014	1.036–1.972

^*^The differences are statistically significant if *P* < 0.05.

## Discussion

Central to the decision of whether to treat a patient with IH is an evaluation of the risks and benefits of each potential therapy. Although widely accepted treatment guidelines for IHs have been published^8^, there is no formula or algorithm that could easily addresses all the factors in this decision. Clinically, treatment decision making should be based on the age and tolerance of the patient; the growth potential, location, and size of the tumor or tumors; the severity of the complication; and the urgency of therapy^1,9^.

A rapid change in the hemangioma lesion is usually noticed within 24 hours following the administration of propranolol, including decreased redness and softening^10^. The therapeutic effect of propranolol is thought to originate from a vasoconstrictive effect on the vascular pericytes in the IH^11–13^. Propranolol also inhibits vasculogenesis and angiogenesis via decreasing the expression of VEGF^14–17^. Other proposed mechanisms are that β-blockers may disrupt the hemodynamic force-induced cell survival and inactivate the rennin-angiotensin system^12,18–20^.

Generally, oral propranolol for the treatment of IH at a dose of up to 2 mg/kg per day, divided into 2 or 3 administrations daily, appears to be well-tolerated in the majority of patients. However, a range of diarrhea, sleep disturbances, peripheral coldness and agitation was reported in the literature. In addition, serious side effects such as bronchospasm/bronchial hyperreactivity, hypoglycemia and persistent hypotension have also been identified during propranolol treatment^3^. In some cases, either temporary or permanent discontinuation of propranolol was required. Unfortunately, rebound growth of IH can occur in up to 25% of patients^21^. Discontinuation of propranolol without tapering would have a significantly increased risk of rebound growth. In addition, as IH growth cessation typically occurs by 9 months of age^22^, propranolol discontinuation at a young age (before 9 -months) has a higher risk of rebound^21^. In the present study, we were not surprised by our observation that 9 patients had a rebound of IH shortly after sudden propranolol withdrawal. Therefore, for patients whose hemangiomas are severe and associated with a high risk of complications or permanent disfigurement, we recommend that the following therapy be administered as early as possible to avoid potential complications.

Recently, we and others reported that oral atenolol was effective for the treatment of problematic IHs^23–26^. Unlike propranolol, atenolol is hydrophilic and does not cross the blood-brain barrier. Theoretically, atenolol could be associated with fewer CNS side-effects^4^. Compared to propranolol, atenolol has a longer terminal half-life of 6–8 hours and therefore can be administered only once daily, which may improve parents’ therapeutic adherence and ensure the efficacy of the treatment. In the present study, we found that a treatment switch from propranolol to atenolol did not compromise the efficacy of therapy.

Our data revealed that ISEs more commonly occurred in younger patients and patients with lower body weight. Sleep disturbance and agitation are generally considered to be side effects attributable to the lipophilic character of propranolol. In this regard, there is evidence suggesting that the blood-brain barrier in young infants is immature and selectively permeable and differs substantially from that of adults; this difference may facilitate the penetration of propranolol^27^. In addition, recent studies demonstrated that young infants who have a history of apnea or neonatal pneumonia appear to be at higher risk for bronchial hyperreactivity after the initiation of β-blocker treatment^28,29^. Bronchial hyperreactivity is a direct effect of non-selective β-blockers (e.g., prapranolol) due to β_2_-AR blockade. Therefore, it seems that hydrophilic, selective β_1_-AR blockers such as atenolol may be useful in treating patients who discontinue propranolol therapy due to these ISEs. In the present study, the patients tolerated the atenolol therapy well. They did not report similar ISEs that were experienced during propranolol treatment. These observations, together with the work presented here, suggest that atenolol may be used as an alternative for the treatment of these potentially high-risk infants.

Young infants, especially preterm neonates and young infants, appear to be at higher risk for propranolol-induced hypoglycemia because they have lower glycogen stores and higher glucose utilization rates. In the literature, there is evidence that propranolol should be used with caution in patients with poor oral intake because these patients may be vulnerable to hypoglycemia^30^. In our study, the two patients who developed hypoglycemia had not been feeding normally. Consistent with previous reports, our patients who developed hypoglycemia were prescribed a relatively low dose of propranolol (2.0 mg/kg/day), further supporting the concept that hypoglycemia associated with propranolol therapy may not be dose-dependent^3,30^. In addition, parent education regarding the proper use of β-blockers is of paramount importance to avoid hypoglycemia^31^. In this regard, parents should be instructed to ensure that their child is fed regularly and to avoid prolonged fasts during treatment. Given that atenolol was administered only once daily, we propose that atenolol be administered during the morning within a half hour after the patients are fed.

Corticosteroids have played an important role in IH management over the past few decades. However, various complications such as growth disorders, stomach irritation and behavioral changes are commonly reported^32^. Nonetheless, corticosteroids remain useful in certain situations, particularly in patients with contraindications to β-blocker treatment (e.g., sinus bradycardia and bronchial asthma). Most recently, two small, randomized control studies demonstrated that propranolol was not inferior to prednisolone with respect to its efficacy in the treatment of IHs, although the findings regarding drug safety were controversial^33,34^. In the present study, we successfully treated IHs with prednisolone as second-line therapy after the failure of propranolol therapy in 3 patients.

Several studies have demonstrated that other β-blockers including nadolol and acebutolol were also effective for the treatment of IHs^35–37^. Recently, the potential efficacy of captopril and itraconazole to treat IHs has also been reported^38,39^. However, there are limited data available regarding the efficacy and safety of these drugs compared to propranolol. Interestingly, sirolimus, an inhibitor of the mammalian target of rapamycin (mTOR), has been successfully used in the treatment of patients with PHACE syndrome (posterior fossa malformations, hemangiomas, arterial malformations, coarctation of the aorta and other cardiac defects, and eye anomalies)^40^. Other anti-hemangioma agents may prove to be more effective in the future. Nonetheless, it is important to proceed cautiously with clinical trials when implementing new therapies in pediatric patients.

## Conclusion

In conclusion, ISEs during the use of propranolol are rare but can be life-threatening. Our study reveals that younger age and lower body weight are independent risk factors for ISEs in patients receiving propranolol therapy. In addition, our data demonstrate that switching from propranolol to atenolol or prednisolone can prevent recurrence of ISEs due to propranolol (not prevent the event themselves), while preserving treatment efficacy.
